# Effects of Antibiotic Cycling Policy on Incidence of Healthcare-Associated MRSA and *Clostridioides difficile* Infection in Secondary Healthcare Settings

**DOI:** 10.3201/eid2501.180111

**Published:** 2019-01

**Authors:** Geraldine Mary Conlon-Bingham, Mamoon Aldeyab, Michael Scott, Mary Patricia Kearney, David Farren, Fiona Gilmore, James McElnay

**Affiliations:** Queen’s University Belfast, Belfast, Northern Ireland, UK (G.M. Conlon-Bingham, J. McElnay);; Antrim Hospital, Antrim, Northern Ireland, UK (M. Aldeyab);; Ulster University, Coleraine, Northern Ireland, UK (M. Aldeyab);; Northern Health and Social Care Trust, Antrim (M. Scott, M.P. Kearney, D. Farren, F. Gilmore)

**Keywords:** Clostridioides difficile, Clostridium difficile, methicillin-resistant Staphylococcus aureus, extended-spectrum β-lactamase, MRSA, CDI, ESBL, antibiotic cycling, nosocomial infections, healthcare-acquired infections, antimicrobial drug resistance, Ireland, bacteria

## Abstract

This quasi-experimental study investigated the effect of an antibiotic cycling policy based on time-series analysis of epidemiologic data, which identified antimicrobial drugs and time periods for restriction. Cyclical restrictions of amoxicillin/clavulanic acid, piperacillin/tazobactam, and clarithromycin were undertaken over a 2-year period in the intervention hospital. We used segmented regression analysis to compare the effect on the incidence of healthcare-associated *Clostridioides difficile* infection (HA-CDI), healthcare-associated methicillin-resistant *Staphylococcus aureus* (HA-MRSA), and new extended-spectrum β-lactamase (ESBL) isolates and on changes in resistance patterns of the HA-MRSA and ESBL organisms between the intervention and control hospitals. HA-CDI incidence did not change. HA-MRSA incidence increased significantly in the intervention hospital. The resistance of new ESBL isolates to amoxicillin/clavulanic acid and piperacillin/tazobactam decreased significantly in the intervention hospital; however, resistance to piperacillin/tazobactam increased after a return to the standard policy. The results question the value of antibiotic cycling to antibiotic stewardship.

Restrictive antimicrobial prescribing guidelines have successfully reduced the incidence of *Clostridioides difficile* infection (CDI; formerly *Clostridium difficile*) and methicillin-resistant *Staphylococcus aureus* (MRSA) (*1*–*6*). However, these guidelines have been suggested to create an environment of antimicrobial homogeneity that may enhance the development and spread of antimicrobial resistance (*7*,*8*). Antibiotic cycling has been proposed as an effective strategy to increase antimicrobial heterogeneity and decrease the development of antimicrobial resistance (*8*,*9*). This method of increasing antimicrobial heterogeneity has had mixed effects on antimicrobial resistance; however, investigations have been conducted mainly in intensive care units (ICUs) and in patients with specific infections (neutropenic sepsis, ventilator-associated pneumonia), and cycling periods have been arbitrarily defined, ranging from 1 week to 6 months (*10*–*22*). In a meta-analysis of studies investigating antibiotic cycling, the optimal cycling period was identified as 30 days (*23*). When the cycling period is too long, the effect becomes equivalent to continuous use of a single agent, increasing antimicrobial homogeneity.

We aimed to evaluate the effect of an antibiotic cycling policy, derived using time-series analysis of retrospective epidemiologic data, on the incidence of healthcare-associated MRSA (HA-MRSA) and healthcare-associated CDI (HA-CDI). A secondary aim was to evaluate the effect of this policy on the incidence of infections caused by extended-spectrum β-lactamase (ESBL)–producing organisms.

## Methods

We conducted the main intervention involving antibiotic cycling in Antrim Area Hospital (AAH), a 447-bed district general teaching hospital in Northern Ireland. Comparative data were collected in Causeway Hospital (CH), a 213-bed district general teaching hospital in Northern Ireland. Both hospitals form part of the Northern Health and Social Care Trust (NHSCT), which serves 436,000 persons. AAH and CH contain general medical, surgical, maternity, and gynecology wards and ICU departments (AAH ICU, 8 beds; CH ICU, 6 beds). AAH also contains a pediatric ward, a neonatal ICU, and a hematology/oncology ward and provides outpatient chemotherapy and renal dialysis services. Other specialist services, such as burn and transplant units, are provided on a regional basis by a neighboring trust. The Office of Research Ethics Committees of Northern Ireland (reference no. 11/NI/0110) and the NHSCT Research Governance Committee (reference no. 10-0219/11) provided ethics approval for this project.

### Study Design

**Figure 1:**
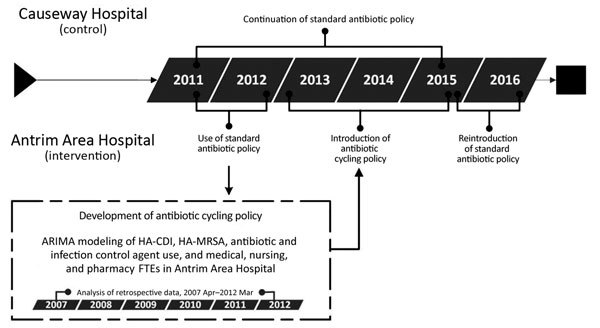
Investigation of the effects of an antibiotic drug cycling policy on the incidence of HA-MRSA and HA-CDI in 2 hospitals, Northern Ireland, UK. ARIMA, autoregressive integrated moving average; HA-CDI, healthcare-associated *Clostridioides difficile* infection; HA-MRSA, healthcare-associated methicillin-resistant *Staphylococcus aureus*; FTE, full-time equivalent.

**Figure 2:**
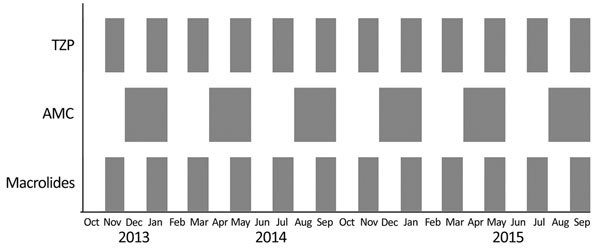
Antibiotic cycling schedule in Antrim Area Hospital, Northern Ireland, UK, showing the months where each antibiotic was recommended. Restrictions in the use of each antibiotic were in place during all other times. AMC, amoxicillin/clavulanic acid; TZP, piperacillin/tazobactam.

The study comprised 3 phases. First was development of an antibiotic cycling policy using retrospective data for April 2007–March 2012; second, implementation and assessment of the effect of this policy in AAH and comparative data collected in CH, where the policy was not implemented; and third, postintervention follow-up after return to the standard antibiotic policy in AAH (Table 1; Figures 1, 2).

**Table 1:** Overview of a study on the effects of an antibiotic drug cycling policy on the incidence of HA-MRSA and HA-CDI in 2 hospitals according to the Orion Statement, Northern Ireland, UK*

Variable	Definition
Population characteristics	The NHSCT is 1 of 5 Health and Social Care Trusts in Northern Ireland, serving ≈436,000 persons. The NHSCT has 2 acute care hospitals: AAH (intervention hospital), containing 447 beds, and CH (control hospital) containing 213 beds. These hospitals provide acute medical, surgical ICU, neonatal, pediatric, and maternity services for the NHSCT. Study wards comprised all adult inpatient wards; ICU, NNU, pediatric, and palliative care wards were excluded.
Retrospective study, 2007 Apr–2012 Mar	The intervention design was as follows: An antibiotic cycling policy was devised based on results of an analysis of HA-CDI and HA-MRSA incidence in AAH during April 2007–March 2012. This analysis identified macrolides and TZP as significantly associated with HA-MRSA with lag times of 1 mo. AMC was identified as significantly associated with HA-CDI with a lag time of 2 mo. Consequently, an antibiotic cycling policy was implemented in AAH that restricted the use of TZP and macrolides in alternate months, and AMC was restricted for 2 consecutive months in every 4 months over a 2-year period.
Comparison of effect of antibiotic cycling policy between AAH and CH, 2011 Nov–2016 Sep†	Comparison of outcome measures before and after the introduction of an antibiotic cycling policy in AAH and between the intervention hospital (AAH) and control hospital (CH). Reintroduction of standard antibiotic policy in AAH during October 2015–September 2016 to determine whether any effect observed during the intervention period was reversed upon return of the standard policy. Comparison of outcome measures between intervention and postintervention periods occurred for AAH only.
General infection control measures	Chlorine dioxide 275 ppm was used for routine environmental decontamination through the study period in both hospitals. Monthly environmental cleanliness audits were conducted on all wards. Throughout the intervention period, infection control practices did not change.
Isolation and elimination policy	All patients in whom CDI was diagnosed were placed in an isolation room. Patients identified as colonized or infected with MRSA were placed in an isolation room when one was available. However, in the event of a shortage of these rooms, these patients were placed in cohort bays.
MRSA admission screening	In both hospitals all patients with a history of MRSA; admitted from a residential or nursing home; admitted from another hospital; admitted to the ICU, NNU, or renal unit; and oncology patients were screened.
Antibiotic stewardship activities	After a CDI outbreak in 2008, restrictions were put in place throughout the NHSCT regarding use of fluoroquinolones, cephalosporins, clindamycin, and carbapenems (*4*). All requests for restricted antibiotic drugs are reviewed by the antimicrobial pharmacists and consultant microbiologists. Weekly audits were conducted on adherence to empirical antibiotic guidelines on all wards.
Definitions	1. HA-CDI incidence: No. patients presenting with CDI >48 h after admission to AAH or CH or any patient presenting with CDI <48 h after admission to these hospitals who had an admission to the same hospital in the preceding 4 wks (*24*).
	2. Other CDI incidence: No. patients presenting with CDI <48 h from admission with no admission to AAH or CH in the preceding 4 wks.
	3. HA-MRSA incidence: No. patients who tested negative or were not screened for MRSA on admission but tested positive for MRSA >48 h after admission (*24*). Each patient was counted once per admission.
	4. Other MRSA incidence: No. patients who tested positive for MRSA <48 h after admission.
	5. New ESBL incidence: No. newly identified patients from whom an ESBL-producing organism was isolated or known patients from whom a new ESBL strain was isolated. Each patient was counted once per admission
	6. Resistant patterns (MRSA and ESBL): No. isolates per month. Duplicate isolates identified within 7 d were excluded.

*Based on (*25*). AAH, Antrim Area Hospital; AMC, amoxicillin/clavulanic acid; CDI, *Clostridioides difficile* infection; CH, Causeway Hospital; ESBL, extended-spectrum β-lactamase; HA, healthcare-associated; ICU, intensive care unit; MRSA, methicillin-resistant *Staphylococcus aureus*; NHSCT, Northern Health and Social Care Trust; NNU, neonatal unit; TZP, piperacillin/tazobactam. †Preintervention period, 2011 Nov–2013 Sep; intervention period, 2013 Oct–2015 Sep; postintervention period, 2015 Oct–2016 Sep.

### Data Collection

For the retrospective part of the study (Figure 1), we determined monthly incidence of the output variables (HA-MRSA, HA-CDI) and input variables (other MRSA; other CDI; antimicrobial and infection control agent use; pharmacy, medical, and nursing staffing levels [full-time equivalents (FTEs), where 1 FTE equals 1 full-time worker]; and age-adjusted Charlson comorbidity index of all patients discharged from AAH). We obtained a record of all MRSA-positive patients identified in AAH from the hospital microbiology laboratory. Cases were classified as HA-MRSA or other MRSA (Table 1). MRSA isolates from clinical samples and screening swab samples were detected according to routine microbiological procedures (*26*). Throughout the study period, targeted MRSA screening was undertaken on admission to AAH or CH for oncology patients; patients with a history of MRSA; patients admitted from a residential or nursing home; and patients admitted to the ICU, neonatal unit, or renal unit. For each patient, repeat isolates during the same admission period were excluded.

We obtained data on the number of patients in whom CDI was diagnosed from the microbiology laboratory and identified HA-CDI and other CDI cases (Table 1). During March 2007–April 2009, we identified *C. difficile* through detection of toxins A and B from the feces of patients with diarrhea using the PremierToxin A and B kit (Meridian Bioscience, Europe, http://www.meridianbioscience.eu) and an ELISA technique. In May 2009, this identification process was changed to a 3-step technique using the Techlab C.diff Quik Chek complete (Abbott, https://www.alere.com/) combined glutamate dehydrogenase and toxin A and B enzyme immunoassay and the Cepheid Xpert C.difficile PCR (http://www.cepheid.com/) for detection of potentially toxigenic strains (*27*).

We obtained records of monthly systemic antimicrobial drug use from the pharmacy computer system and converted to defined daily doses (DDD) (*26*,*28*). The use of antiseptic agents, such as chlorhexidine skin wash (liters), alcohol-impregnated wipes (number of wipes), and alcohol-based hand rub (liters), acted as a proxy measure for hand hygiene in the study hospitals (*29*). We determined monthly use of these agents across both hospitals during the study period from the pharmacy computer system and the National Health Service Supply Chain. We obtained the monthly number of nursing, auxiliary nursing, clinical pharmacy, and medical FTEs in AAH from the NHSCT Finance Department. We determined the monthly average age-adjusted comorbidity for AAH by calculating the Charlson age-adjusted comorbidity index of all patients discharged each month (*30*). For all of these variables, we adjusted the value to an incidence or use per 100 occupied bed days (OBD).

Using linear regression, we determined the trends in each of the explanatory variables. We constructed autoregressive integrated moving average models to model the relationship between the input and output variables in AAH for April 2007–March 2012 (Appendix). Based on the antimicrobial drugs that were significantly associated with HA-CDI and HA-MRSA, together with the time lag between observing a change in the antimicrobial drug use and a corresponding change in HA-MRSA or HA-CDI incidence, we developed an antibiotic cycling policy and implemented it in AAH over a 2-year period (October 13–September 15) (Figure 2).

### Implementation of Cycling Policy

The antibiotic cycling policy was implemented on wards in AAH except pediatrics, neonatal unit, palliative care ward, and ICU. We excluded the palliative care ward and ICU because patients in these departments usually have received multiple previous courses of antimicrobial drugs; therefore, empirical guidelines do not apply, and antimicrobial therapy is guided by advice from the NHSCT Consultant Microbiologists. The cycling policy affected the antimicrobial recommendations for community-acquired pneumonia, hospital-acquired pneumonia, intraabdominal sepsis, and urinary tract infections. In months during which all 3 antimicrobial drugs (amoxicillin/clavulanic acid [AMC], piperacillin/tazobactam [TZP], and macrolides) were restricted, levofloxacin was recommended for the treatment of severe community-acquired pneumonia and intraabdominal sepsis. However, levofloxacin could be supplied only on receipt of an order form signed by medical staff. All patients prescribed levofloxacin were followed up for 12 weeks. During this time, the NHSCT laboratory system was used to determine the proportion of patients from whom *C. difficile* was isolated from fecal samples or MRSA from screening swab samples and clinical samples. We constructed life tables to determine the probability of remaining CDI- and MRSA-free for each week after levofloxacin treatment. After this, the cumulative probability of remaining CDI- or MRSA-free was determined and expressed as a percentage.

Before implementation of this policy, clinical staff were trained on the intervention. Monthly reminders about the policy changes were sent to staff groups, and each month a new antibiotic policy was displayed on participating wards.

During periods of restriction of a cycled antimicrobial drug, stock was removed from all bulk orders, and these drugs could be supplied only on receipt of a request form signed by a clinician, outlining the patient’s details and indication for treatment. During this time, the Trustwide antibiotic stewardship activities continued as usual. These included review of requests of all restricted antimicrobial drugs by the pharmacist and consultant microbiologist and weekly audits on adherence to the Trust antibiotic policy. Where inappropriate use was identified, it was fed back to the prescriber in real time and monthly to all consultants and the medical director.

For the preintervention (November 11–September 13), intervention (October 13–September 15), and postintervention (October 15–September 2016) periods in AAH, the incidence of HA-CDI, other CDI, HA-MRSA, other MRSA, and the use of antimicrobial drugs and infection control agents were collected as described. Furthermore, HA-MRSA resistance patterns and new ESBL incidence and resistance patterns were determined. These variables were also identified in CH (November 2011–September 2015) for comparative analysis. For HA-MRSA isolates identified from clinical samples, we determined the percentage change in ciprofloxacin, erythromycin, AMC, and TZP resistance during the preintervention and intervention periods for both hospitals. Duplicate isolates identified within 7 days of the initial isolate were excluded.

We identified ESBL isolates from clinical samples using the method described by the European Committee on Antimicrobial Sensitivity Testing for the detection of ESBLs (*31*). The monthly number of new ESBL isolates and percentage change in gentamicin, ciprofloxacin, AMC, and TZP resistance during the preintervention and intervention periods was determined for both hospitals.

We assessed the effect of the antibiotic cycling policy on HA-MRSA, HA-CDI, and new ESBL incidence in AAH using segmented regression analysis (*32*). Segmented regression analysis was used to identify changes in the outcome measures in CH during the study period. We examined the effect of the antibiotic cycling policy on changes in HA-MRSA and new ESBL resistance patterns in AAH in comparison with CH using χ^2^ analysis.

For AAH only, we used the aforementioned techniques to determine the effect of reverting to the standard antibiotic policy on HA-MRSA, HA-CDI, and new ESBL incidence during October 2015–September 2016. We also determined changes in resistance patterns of HA-MRSA and new ESBL isolates.

For all analyses, we considered p<0.05 to be statistically significant. All analyses were conducted using Eviews 8 software (QMS, http://www.eviews.com/home.html) and Microsoft Excel version 13 (https://www.microsoft.com/en-us).

## Results

### Retrospective Analysis

During April 2007–March 2012, a total of 275 cases of HA-CDI (average monthly incidence 0.047 cases/100 OBD [range 0–0.18 cases/100 OBD]) and 653 cases of HA-MRSA (average monthly incidence 0.11 cases/100 OBD [range 0.03–0.21 cases/100 OBD]) were reported in AAH (Appendix, Figures 1, 2). Temporal variations in HA-CDI incidence followed temporal variations in fluoroquinolone use (coefficient 0.005, p<0.01), with a 1-month lag time, and AMC use (coefficient 0.0004, p = 0.02; Appendix Table 2), with a 2-month lag time. We identified significant temporal associations between HA-MRSA incidence and fluoroquinolone use (coefficient 0.004, p<0.01), with a 3-month lag time; macrolide use (coefficient 0.002, p <0.01), with a 1-month lag time; and TZP use (coefficient 0.010, p<0.01), with a 1-month lag time (Appendix Table 3). Based on these findings, an antibiotic cycling policy was implemented in AAH during October 2013–September 2015 (Figure 2).

### Evaluation of Effect of Intervention

**Figure 3:**
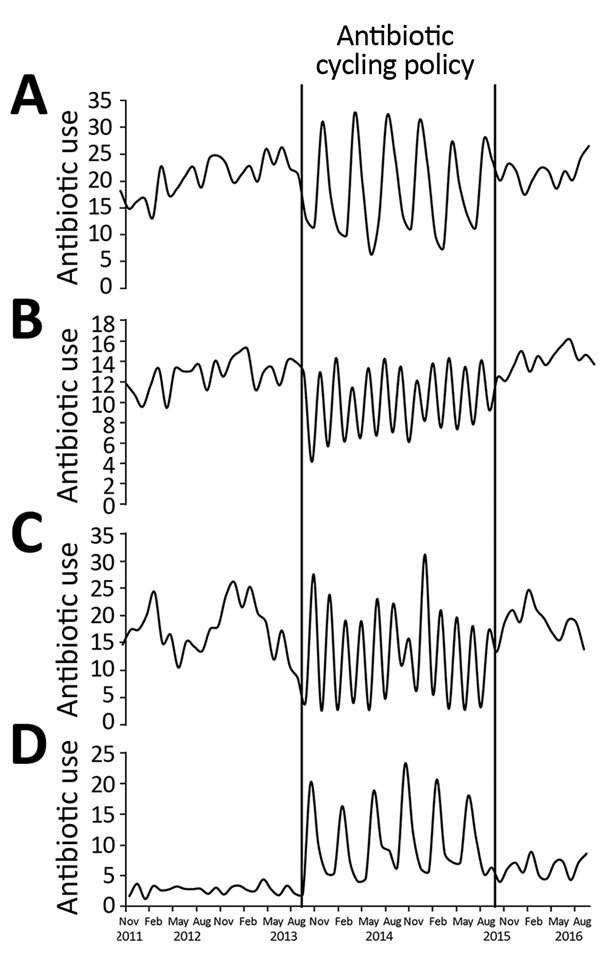
Trends in antibiotic use throughout preintervention (November 2011–September 2013), intervention (October 2013–September 2015), and postintervention (October 15–September 2016) periods in Antrim Area Hospital, Northern Ireland, UK. A) amoxicillin/clavulanic acid; B) piperacillin/tazobactam; C) macrolides; D) fluoroquinolones. Antibiotic is defined daily doses/100 bad days.

During the preintervention period, AMC use increased significantly in AAH (coefficient 0.3603, p<0.01) and CH (coefficient 0.3848, p = 0.01) (Appendix Table 4). In AAH, we observed a significant increase in TZP (coefficient 0.1119, p = 0.02), penicillins with extended-spectrum (coefficient 0.4702, p = 0.02), and first-generation cephalosporins (coefficient 0.022, p<0.01) during this period. In addition, a borderline significant increase in total antimicrobial drug use was evident. During the intervention period in both hospitals, no change in the overall use of TZP (AAH coefficient 0.1211, p = 0.25; CH coefficient 0.086, p = 0.16), AMC (AAH coefficient 0.1212, p = 0.64; CH coefficient 0.0056, p = 0.95), and macrolide (AAH coefficient −0.0126, p = 0.96; CH coefficient 0.0756, p = 0.5272) was observed. Despite this, in AAH, the monthly use of these antimicrobial drugs followed the trend that would be expected during the use of the antibiotic cycling policy (Figure 3). In AAH, during the intervention period, use of β-lactamase–sensitive penicillins (coefficient −0.1171, p<0.01) and carbapenems (coefficient −0.1124, p<0.01) decreased significantly. In CH during the intervention period, use of penicillins with extended-spectrum (coefficient 0.7081, p<0.01), tetracyclines (coefficient 0.3596, p<0.01), and total antimicrobial drugs (coefficient 1.5278, p<0.01) increased significantly. Use of carbapenems, first-generation cephalosporins, and β-lactam–sensitive penicillins also decreased significantly (Appendix Table 4).

**Figure 4:**
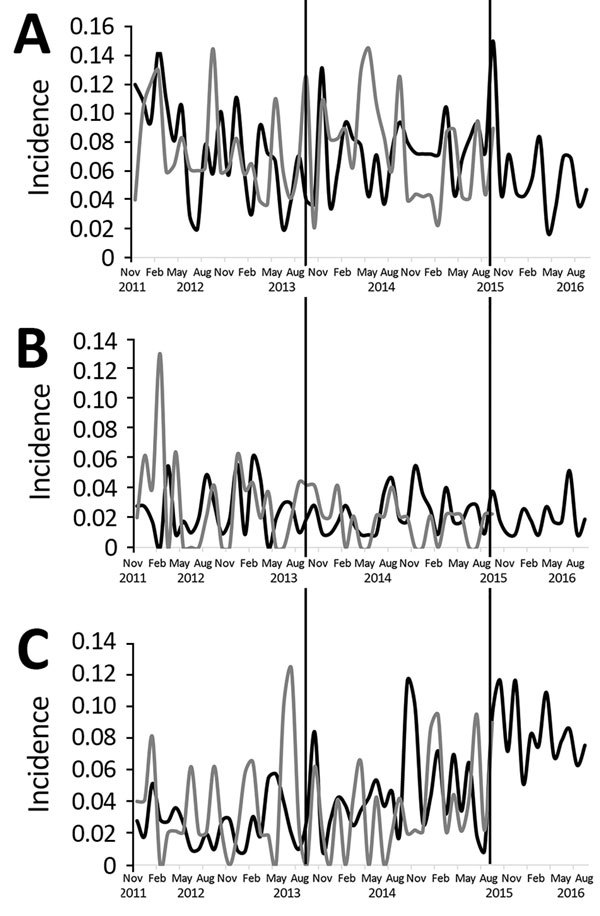
Incidence of healthcare-associated methicillin-resistant *Staphylococcus aureus* (A), healthcare-associated *Clostridium difficile* infection (B), and new extended-spectrum β-lactamase (C) cases throughout preintervention (November 2011–September 2013), intervention (October 2013–September 2015), and postintervention (October 15–September 2016) periods in Antrim Area Hospital and Causeway Hospital, Northern Ireland, UK. Black lines, Antrim Area Hospital; gray lines, Causeway Hospital. Incidence is number of cases per 100 occupied bed days.

Throughout the preintervention period, the average monthly adherence to the standard antibiotic policy was 92% in AAH and 91% in CH. HA-CDI and new ESBL incidence did not change significantly in either hospital; however, after the antibiotic cycling policy was introduced in AAH, a significant increasing trend in HA-MRSA incidence was observed, which was not evident in CH (Table 2; Figure 4). A subset analysis of the AAH HA-MRSA isolates from clinical samples also demonstrated a significant increase in the HA-MRSA trend (coefficient 0.0023, p<0.01).

**Table 2:** Segmented regression analysis of the incidence of HA-CDI, HA-MRSA, and infections caused by new ESBL isolates in a hospital with a cycling policy and a hospital with a standard policy, Northern Ireland, UK*

Variable	AAH, cycling policy					CH, standard policy			
Coefficient	95% CI	SE	p value	Coefficient	95% CI	SE	p value
HA-CDI									
Constant	0.0236	0.0099 to 0.0373	0.0068	**<0.01**		−2.9873†	−3.669 to −2.3055	0.3378	**<0.01**
Trend	9.34 × 10^−5^	−0.0009 to 0.0011	0.0005	0.85		−0.01905†	–0.0603 to 0.0222	0.0204	0.36
Change									
In level	−0.0084	−0.0270 to 0.0102	0.0092	0.36		−0.1335†	−0.6324 to 0.3654	0.2472	0.59
In trend	0.0004	−0.0010 to 0.0017	0.0007	0.58		0.0053†	−0.0394 to 0.0500	0.0221	0.81
HA-MRSA									
Constant	0.1100	0.0856 to 0.1343	0.0121	**<0.01**		0.0849	0.0562 to 0.1135	0.01421	**<0.01**
Trend	−2.96 × 10^−3^	−0.0047 to −0.0012	0.0009	**<0.01**		−0.0008	−0.0029 to 0.0013	0.0010	0.46
Change									
In level	0.0196	−0.0134 to 0.05261	0.01634	0.24		0.0230	−0.0158 to 0.0618	0.01925	0.24
In trend	0.0041	0.0017–0.0066	0.0012	**<0.01**		−0.0005	−0.0034 to 0.0023	0.0014	0.72
New ESBL									
Constant	−3.7851†	−4.3481 to −3.2222	0.2790	**<0.01**		0.0370	0.0119 to 0.0621	0.0124	**<0.01**
Trend	−0.0018†	−0.0428 to 0.0393	0.0203	0.93		−0.0004	−0.0023 to 0.0015	0.0009	0.68
Change									
In level	0.4846†	−0.2776 to 1.2469	0.3777	0.21		−0.0093	−0.0440 to 0.0045	0.0172	0.59
In trend	0.0099†	−0.0464 to 0.0662	0.0279	0.72		0.0019	−0.0006 to 0.1564	0.0013	0.13
Outlier at 2013 Jul, CH only	NA	NA	NA	NA		0.0949	0.0333 to 0.1564	0.0305	**<0.01**

*Bold indicates statistical significance. AAH, Antrim Area Hospital; CH, Causeway Hospital; CDI, *Clostridioides difficile* infection; ESBL, extended-spectrum β-lactamase; HA, healthcare-associated; MRSA, methicillin-resistant *Staphylococcus aureus*; NA, not applicable.  †Data logarithmically transformed.

During the intervention period, 792 patients received levofloxacin; 15 tested MRSA positive before receiving levofloxacin and were excluded from the MRSA cohort (Appendix Figure 3). Eleven patients tested MRSA positive during the follow-up period, and 81 patients died. The probability of remaining MRSA-free after the 12-week follow-up period was 98.5% (Appendix Figure 4). The CDI cohort comprised 792 patients, of whom 87 died during the follow-up period. Two patients tested *C. difficile* glutamate dehydrogenase–positive and *C. difficile* toxin negative, and 9 patients tested *C. difficile* toxin positive (Appendix Figure 5). The probability of remaining free of CDI during the 12-week follow-up period was 98.5% (Appendix Figure 6).

### Evaluation of Postintervention Effect

During the postintervention period, the average monthly adherence to the standard antibiotic policy in AAH remained at 92% (Appendix Table 4). The overall trend in the use of each of the cycled antimicrobial drugs remained unchanged during the postintervention period (TZP trend coefficient 0.147, p = 0.12; macrolide trend coefficient −0.1699, p = 0.55; AMC trend coefficient 0.2996, p = 0.16; fluoroquinolone trend coefficient 0.148, p = 0.30). However, in AAH, use of monobactams decreased significantly (coefficient −0.2382, p<0.01 [Appendix Table 4]). Segmented regression analysis identified an immediate borderline significant decrease in HA-MRSA incidence and an immediate significant increase in new ESBL incidence (Table 3). A subset analysis of AAH HA-MRSA isolates from clinical samples demonstrated no change in the incidence of this organism after the end of antibiotic cycling; however, the level of HA-MRSA from clinical samples immediately decreased significantly (coefficient −0.029, p = 0.0261).

**Table 3:** Segmented regression analysis of effect of stopping cycling on the incidence of HA-CDI, HA-MRSA, and new ESBL isolates in Antrim Area Hospital, Northern Ireland, UK*

Variable	Coefficient	95% CI	SE	p value
HA-CDI				
Constant	0.0173	0.0062 to 0.0284	0.0054	**<0.01**
Trend	4.73 × 10^−4^	−0.0003 to 0.0012	0.0004	0.22
Change				
In level	−0.0159	−0.0352 to 0.0033	0.0094	0.10
In trend	0.0005	−0.0018 to 0.0028	0.0011	0.65
HA-MRSA				
Constant	0.0598	0.0388 to 0.0810	0.0102	**<0.01**
Trend	0.0012	−0.0002 to 0.0027	0.0007	0.10
Change				
In level	−0.0350	−0.0711 to −0.001	0.0179	**0.0**6
In trend	−0.0016	−0.002 to 0.0027	0.0022	0.45
New ESBL				
Constant	0.0438	0.0200 to 0.0672	0.0115	**<0.01**
Trend	0.0005	−0.0011 to 0.0021	0.0008	0.56
Change				
In level	0.0419	0.0017 to 0.0820	0.0199	**0.04**
In trend	−0.0026	−0.0074 to 0.0023	0.0024	0.30

*In this hospital, a cycling policy was in place during October 2013–September 2015, and a standard policy was in place during October 2015–September 2016. Bold indicates statistical significance. ESBL, extended-spectrum β-lactamase; HA-CDI, healthcare-associated *Clostridioides difficile* infection; HA-MRSA, healthcare-associated methicillin-resistant *Staphylococcus aureus*.

### Effect of Antibiotic Cycling on Antimicrobial Resistance

During the preintervention, intervention, and postintervention periods in AAH and the equivalent periods in CH, HA-MRSA resistance to ciprofloxacin and erythromycin did not change (Table 4). We observed a significant decrease in resistance to AMC (–31.85% change, p<0.01) and TZP (–54.79% change, p<0.01) among new ESBL isolates in AAH during the intervention period (Table 5). In AAH after reintroduction of the standard antibiotic policy, new ESBL resistance to TZP (+11.75% change, p = 0.04), gentamicin (+23.5% change, p<0.01), and ciprofloxacin (+16.32% change, p<0.01) increased significantly.

**Table 4:** Changes in resistance patterns of healthcare-associated methicillin-resistant *Staphylococcus aureus* clinical isolates, Northern Ireland, UK*

Antibiotic comparison	AAH, no. resistant isolates/total tested (%)				CH, no. resistant isolates/total tested (%)		
Preintervention resistance	Intervention resistance	p value	Preintervention resistance	Intervention resistance	p value
Preintervention vs. intervention							
Amoxicillin/clavulanic acid	2/2 (100)	2/2 (100)	NA		2/2 (100)	0	NA
Piperacillin/tazobactam	1/1 (100)	1/1 (100)	NA		1/1 (100)	0	NA
Ciprofloxacin	66/66 (100)	77/78 (98.7)	0.99		31/31 (100)	33/34 (97.1)	0.17
Erythromycin	54/70 (77.1)	69/80 (86.3)	0.18		23/32 (71.9)	22/35 (65.9)	0.43
Intervention vs. postintervention							
Amoxicillin/clavulanic acid	2/2 (100)	2/2 (100)	NA		NR	NR	NR
Piperacillin/tazobactam	1/1 (100)	0	NA		NR	NR	NR
Ciprofloxacin	77/78 (98.7)	27/27 (100)	0.74		NR	NR	NR
Erythromycin	69/80 (86.3)	23/28 (82.1)	0.40		NR	NR	NR

*In AAH (antibiotic cycling policy) and CH (standard antibiotic policy), the preintervention period was November 2011–September 2013, and the intervention period was October 2013–September 2015. In AAH, the postintervention period was October 2015–September 2016. AAH, Antrim Area Hospital; CH, Causeway Hospital; NA, no isolates tested against amoxicillin/clavulanic acid and piperacillin/tazobactam; NR, data not recorded.

**Table 5:** Changes in resistance patterns of new extended-spectrum β-lactamase isolates during the preintervention period, Northern Ireland, UK*

Antibiotic comparison	AAH, no. resistant isolates/total tested (%)				CH, no. resistant isolates/total tested (%)		
Preintervention resistance	Intervention resistance	p value		Preintervention resistance	Intervention resistance	p value
Preintervention vs. intervention							
Amoxicillin/clavulanic acid	59/65 (90.8)	76/129 (58.9)	**<0.0**1		38/41 (92.7)	33/42 (78.6)	0.07
Piperacillin/tazobactam	50/65 (76.9)	29/131 (22.1)	**<0.0**1		28/41 (68.3)	21/41 (51.2)	0.11
Ciprofloxacin	43/66 (65.2)	78/132 (59.1)	0.41		25/40 (62.5)	26/40 (65.0)	0.82
Gentamicin	20/67 (29.9)	38/135 (28.1)	0.81		15/41 (36.6)	22/42 (52.4)	0.15
Intervention vs. postintervention							
Amoxicillin/clavulanic acid	76/129 (58.9)	80/122 (65.6)	0.28		NR	NR	NR
Piperacillin/tazobactam	29/131 (22.1)	41/121 (33.9)	**0.04**		NR	NR	NR
Ciprofloxacin	78/132 (59.1)	92/122 (75.4)	**<0.01**		NR	NR	NR
Gentamicin	38/135 (28.1)	63/122 (51.6)	**<0.01**		NR	NR	NR

*In AAH (antibiotic cycling policy) and CH (standard antibiotic policy), the reintervention period was November 2011–September 2013, and the intervention period was October 2013–September 2015. In AAH, the postintervention period was October 2015–September 2016. Bold indicates statistical significance. AAH, Antrim Area Hospital; CH, Causeway Hospital; NR, data not recorded.

## Discussion

The use of antibiotic cycling to reduce antimicrobial resistance has been heavily debated, and many studies have produced conflicting results (*10*–*22*,*33*). We aimed to implement an antibiotic cycling policy throughout a hospital where the analysis of local epidemiologic data using autoregressive integrated moving average modeling provided the framework for the antibiotic cycling policy. The cycling of AMC, TZP, and macrolides, which the initial retrospective study predicted would decrease the incidence of HA-CDI and HA-MRSA, did not achieve this objective. HA-CDI incidence did not change during the intervention period. In addition, for patients who received levofloxacin, the probability of remaining free from CDI or MRSA in the 12 weeks after treatment was 98.5%. The incidence of new ESBL isolates during the intervention period remained unchanged, but resistance of new ESBL isolates to TZP and AMC decreased. During the postintervention period, when the standard antibiotic policy was in place, resistance of ESBL-producing organisms to TZP increased.

During the antibiotic cycling period, the incidence of HA-MRSA from clinical and surveillance samples increased significantly in AAH but reversed when the standard antibiotic policy was reintroduced. Many studies have highlighted the increased risk for MRSA infection in MRSA carriers. Recent studies reported an increased risk for invasive MRSA infection in persistent and intermittent MRSA carriage compared with placebo (*34*–*36*). The findings in our study are supported by those of a subset analysis of HA-MRSA isolates from clinical samples but must be interpreted with caution because of the small number of clinical samples included in the total HA-MRSA sample.

A recent meta-analysis hypothesized that a 1-month cycling period is the optimum time frame to reduce antimicrobial resistance (*23*). In our study, the monthly cycling of clarithromycin and TZP did not decrease the incidence of HA-MRSA and HA-CDI. The reintroduction of fluoroquinolones, which are known to be associated with CDI and MRSA, may have contributed to the increase in HA-MRSA (*4*,*37*,*38*). In CH, fluoroquinolone use (5.86 DDD/100 OBD) was about half that in AAH (10.38 DDD/100 OBD), and HA-MRSA incidence did not change. Despite increased fluoroquinolone use in AAH, HA-CDI incidence did not change during the intervention and postintervention periods, when fluoroquinolone use decreased. Other studies have demonstrated decreases in CDI after reductions in AMC, fluoroquinolones, cephalosporin, clindamycin, and amoxicillin use (*39*,*40*). However, in these studies, the baseline incidence of CDI was ≈10-fold higher than in our investigation (0.2 cases/100 OBD, vs. 0.02 cases/100 OBD in AAH). Furthermore, fluoroquinolones are particularly associated with *C. difficile* ribotype 027 (*4*). During an outbreak of this ribotype in AAH, average monthly fluoroquinolone use was 13 DDD/100 OBD, which was less than the highest levels (23 DDD/100 OBD) during the intervention period in our investigation (*4*). Despite these well-documented associations, we observed no such increase in HA-CDI during the cycling period. Vernaz et al. found no significant association between fluoroquinolone use and CDI in a setting where the baseline incidence was similar to that in AAH (0.027 cases/100 OBD) (*40*). That study suggested that in the nonoutbreak situation with an absence of the 027 strain and low baseline CDI incidence, when coupled with good infection control practices, antimicrobial drugs might play a less important role in transmitting this organism.

Although the antibiotic cycling policy was not designed to reduce ESBL incidence, we monitored this organism to ensure it did not increase as an inadvertent effect of the intervention, particularly the reintroduction of fluoroquinolones. New ESBL incidence did not change during the intervention period, but upon return of the standard policy, new ESBL incidence immediately increased in AAH. Because this increase did not continue throughout the postintervention period, it might have been a delayed effect of the antibiotic cycling policy. An association between ESBL incidence in AAH and both hospital fluoroquinolone use and community AMC use has previously been described; therefore, the increase in new ESBL-producing organisms in our study might have resulted from the increased use of fluoroquinolones during the intervention and postintervention periods (*41*). Primary care antimicrobial drug use also might have contributed to the increase in ESBL-producing organisms, but because we did not measure that variable, we cannot quantify the association. 

Previous studies have demonstrated reversal in fluoroquinolone resistance after continuous restriction of this drug (*41*). However, our study demonstrated a reduction in TZP and AMC resistance after cycling of these agents, which was reversed for TZP upon its return to the policy, suggesting that through cycling, the balance between using TZP and reducing resistance can be achieved. Several studies have demonstrated the effectiveness of β-lactam/β-lactamase combinations in treating infections caused by ESBL-producing organisms, particularly infections with organisms for which the MICs for TZP are <16 μg/L (*42*–*45*). Harris et al. highlighted the lack of well-designed studies proving that β-lactam/β-lactamase combinations are inferior to carbapenems in treating these infections and called for more robust studies in this area (*46*). The findings of our study may provide a treatment option in less severe infections caused by ESBL-producing organisms, enabling carbapenems to be spared.

The successful implementation of the antibiotic cycling policy relied heavily on face-to-face engagement with all hospital staff during its development. We sought feedback from each department about potential issues with the proposed restrictions. A main difficulty was managing the restriction at ward level, ensuring restricted stock could not be unnecessarily prescribed. AAH benefited from the presence of a clinical pharmacist and pharmacy technician on all inpatient wards. These pharmacists were vital in ensuring that all ward staff were aware of the restrictions in a given month. Pharmacy technicians removed restricted antimicrobial drugs from the ward stock lists so the ward could not order a restricted drug without a preorder form outlining the patient’s details and the reason for the prescription. This resource-intense intervention might be challenging to deliver in other settings, particularly where clinical pharmacy resources are limited.

Because this study was ecological in design, it suffers from the ecological fallacy, where inferences made at the population level cannot be extrapolated to the patient level. However, because of the complex dynamics of healthcare-associated infection transmission, antimicrobial drug use at the population level contributes to the individual risk for healthcare-associated infections (*47*). The use of alcohol-based hand rub and chlorhexidine skin wash was used as proxy for infection control practices because obtaining consistent data on compliance with isolation and infection control policies was not possible. During the intervention and postintervention periods, the use of other groups of antibiotics in AAH and CH changed significantly because controlling the use of all antimicrobial drugs was not possible. The decrease in monobactam use in AAH during the postintervention period resulted from ending the intervention, whereby aztreonam was replaced by the previously restricted AMC and TZP.

For analysis of changes in HA-MRSA resistance patterns, we included only clinical samples, which accounted for 30% of total MRSA isolates. Before the intervention, the trend in new ESBL resistance to AMC and TZP was decreasing as a result of a change in interpretative standards from Clinical and Laboratory Standards Institute to European Committee on Antimicrobial Susceptibility Testing breakpoints in November 2011 (*31*). Selection and information biases were limited in this study through the inclusion of all patients with HA-MRSA, HA-CDI, and a new ESBL-producing organism. All data were collected from electronic databases that were populated as part of routine microbiology procedures. This study is also limited by the relatively short intervention period of 2 years, compared with other studies with intervention periods of 5–9 years, which resulted in changes in antimicrobial resistance (*13*,*18*).

Our results suggest that antibiotic cycling is not an appropriate strategy to reduce the incidence of HA-MRSA or HA-CDI but might be effective in reducing ESBL resistance. The increased use of fluoroquinolones in a cyclical fashion was not associated with any increase in HA-CDI, suggesting that this method may help diversify antimicrobial drug use while mitigating adverse effects.

Appendix Autoregressive integrated moving average modeling of input and output variables for study of effects of antibiotic cycling policy on healthcare-associated methicillin-resistant *Staphylococcus aureus* and *Clostridioides difficile* infection in secondary healthcare settings.
