# Supplementary material for: Effects of Antibiotic Cycling Policy on Incidence of Healthcare-Associated MRSA and Clostridioides difficile Infection in Secondary Healthcare Settings
Source: Emerg Infect Dis. 2019 Jan;25(1):52–62. doi: 10.3201/eid2501.180111 (PMC6302607; doi:10.3201/eid2501.180111)
Supplement: Appendix — Autoregressive integrated moving average modeling of input and output variables for study of effects of antibiotic cycling policy on healthcare-associated methicillin-resistant Staphylococcus aureus and Clostridioides difficile infection in secondary healthcare settings. [file 18-0111-Techapp-s1.pdf]

Article DOI: https://doi.org/10.3201/eid2501.180111

Effects of Antimicrobial Cycling Policy on Incidence of Nosocomial MRSA and *Clostridioides difficile* Infection in Secondary Healthcare Settings

Appendix

ARIMA Modelling of Input and Output Variables

Time series analysis models were constructed to evaluate the relationship between the explanatory variables of antibiotic use, infection control agent use, staffing levels and Charlson age-adjusted comorbidity index and the outcome variables of HA-MRSA and HA-CDI. ARIMA models using the Box-Jenkins method for analysis were constructed to evaluate the relationships between the explanatory and outcome variables (*1*). Linear transfer function (LTF) models were then constructed using the method previously described by Aldeyab et al. to identify which of the explanatory variables was significantly associated with HA-MRSA and HA-CDI in Antrim Hospital, while considering any time lags before an effect is observed (*2*).

References

1. Helfenstein U. Box-Jenkins modelling in medical research. Stat Methods Med Res. 1996;5:3–22. PubMed http://dx.doi.org/10.1177/096228029600500102

2. Aldeyab MA, Monnet DL, López-Lozano JM, Hughes CM, Scott MG, Kearney MP, et al. Modelling the impact of antibiotic use and infection control practices on the incidence of hospital-acquired methicillin-resistant *Staphylococcus aureus*: a time-series analysis. J Antimicrob Chemother. 2008;62:593–600. PubMed http://dx.doi.org/10.1093/jac/dkn198

Appendix Table 1. Trends of explanatory variables during April 2007–March 2012*

Variable	Mean (SD)	Coefficient	p value
HA-CDI, no. cases/100 OBD	0.05 (0.04)	-0.0014	**<0.0001**
Other CDI, no. cases/100 OBD	0.03(0.02)	-0.0005	**0.0012**
HA-MRSA, no. cases/100 OBD	0.11 (0.04)	-0.0006	0.0532
Other MRSA, no. cases/100 OBD	0.63 (0.13)	-0.0040	**<0.0001**
All antibiotics, DDDs/100 OBD	116.26 (17.96)	0.6825	**<0.0001**
β-lactamase–sensitive penicillins_J01CE, DDDs/100 OBD	2.24 (0.86)	-0.0006	0.9195
β-lactamase–resistant penicillins_J01CF, DDDs/100 OBD	5.73 (2.14)	0.06937	**<0.0001**
Penicillins with extended spectrum_J01CA, DDDs/100 OBD	13.81 (11.18)	0.5554	**<0.0001**
Co-amoxiclav, DDDs/100 OBD	32.51 (9.83)	-0.4084	**<0.0001**
Piperacillin/tazobactam, DDDs/100 OBD	7.74 (2.27)	0.1161	**<0.0001**
1st-generation cephalosporins_J01DB, DDDs/100 OBD	0.25 (0.19)	-0.0057	**<0.0001**
2nd-generation cephalosporins_J01DC, DDDs/100 OBD	0.49 (1.14)	-0.03540	**<0.0001**
3rd-generation cephalosporins_J01DD, DDDs/100 OBD	0.46 (0.34)	-0.0033	0.1954
Macrolides J01FA, DDDs/100 OBD	19.65 (4.30)	-0.0481	0.1345
Fluoroquinolones_J01MA, DDDs/100 OBD	3.64 (4.07)	-0.1212	**<0.0001**
Tetracyclines_J01A, DDDs/100 OBD	5.48 (4.10)	0.1894	**<0.0001**
Alcohol-based hand rub, L/100 OBD	1.23 (0.40)	0.0136	**<0.0001**
Alcohol-impregnated skin wipes, including alcohol and chlorhexidine wipes, no. wipes/100 OBD	248.65 (35.25)	0.5040	0.0544
Chlorhexidine scrub, L/100 OBD	1.8 (0.3)	-0.02	**0.0001**
Chlorhexidine powder, g/100 OBD	0.66 (0.53)	-0.014	**0.0003**
Gloves, no. gloves/100 OBD	2907.42 (473.32)	7.2200	**0.04**
Age-adjusted co-morbidity, score/100 OBD	2.94 (0.27)	0.0136	**<0.0001**
Pharmacist FTE, FTE/100 OBD	0.40 (0.05)	0.00213	**<0.0001**
Pharmacy technician FTE, FTE/100 OBD	0.35 (0.05)	0.0014	**0.0001**
Nurse FTE, FTE/100 OBD	4.26 (0.26)	0.0093	**<0.0001**
Nursing auxiliary FTE, FTE/100 OBD	1.19 (0.07)	0.0021	**<0.0001**
Medical staff FTE, all grades, consultant to F1 except GPWSI, FTE/100 OBD	1.30 (0.22)	0.0100	**<0.0001**

*CDI, *Clostridiodes difficile* infection; DDDs, defined daily doses; FTE, full-time equivalent; GPWSI, general practitioner with a special interest; HA, healthcare-associated; MRSA, methicillin-resistant *Staphylococcus aureus*; OBD, occupied bed days. Bold type indicates statistical significance.

A**ppendix Table 2.** Time series analysis model for HA-CDI series (R^2 0.75)*

Variable	Lag time, mo.†	Coefficient (95% CI)‡	p value
Constant	NA	–0.007 (–0.019 to 0.005)	0.270
Fluoroquinolones	1	0.005 (0.004–0.006)	**<0.001**
Co-amoxiclav	2	0.0004 (0.0001–0.0007)	**0.016**
CDI outbreak	NA	0.032 (0.022–0.042)	**<0.001**
Other CDI cases	3	0.420 (0.143–0.697)	**0.004**
AR	1	–0.388 (–0.656 to –0.12)	**0.006**
MA	2	–0.551 (–0.802 to –0.30)	**<0.001**

*AR, autoregressive term representing past incidence density of HA-CDI; CDI, *Clostridiodes difficile* infection; HA, healthcare-associated: MA, moving average term representing past disturbances in the incidence density of HA-CDI; NA, not applicable. Bold indicates statistical significance.
†Delay necessary to observe an effect.
‡Size and direction of the effect.

Appendix Table 3. Time series analysis model for HA-MRSA series (R^2 0.48)*

Variable	Lag time, mos.†	Coefficient (95% CI)‡	p value
Constant		0.116 (0.049–0.183)	**0.001**
Fluoroquinolones	3	0.004 (0.003–0.005)	**<0.001**
Macrolide	1	0.002 (0.001–0.003)	**0.001**
Piperacillin/tazobactam	1	0.010 (0.007–0.013)	**<0.001**
Pharmacist FTE	1	–0.373 (–0.526 to –0.22)	**<0.001**
MA[c]	1	–0.671 (–0.905 to –0.437)	**<0.001**

*FTE, full-time equivalent; HA-MRSA, healthcare-associated methicillin-resistant *Staphylococcus aureus*; MA, moving average term representing past disturbances in the incidence density of HA-MRSA. Bold indicates statistical significance.
†Delay necessary to observe an effect.
‡Size and direction of the effect.

Appendix Table 4. Descriptive statistics for input and output variables for the pre-intervention, intervention, and postintervention periods in Antrim and Causeway Hospitals, Northern Ireland, UK*

Variable	Pre-intervention, 2011 Nov–2013 Sep Average use (SD)	Pre-intervention Trend coefficient	Pre-intervention p value	Intervention, 2013 Oct–2015 Sep Average use (SD)	Intervention Trend	Intervention p value	Postintervention, 2015 Oct–2016 Sep Average incidence/use (SD)	Postintervention Trend coefficient	Postintervention p value
Antrim Hospital									
HA-CDI, no. cases/100 OBD	0.025 (0.018)	9.34×10^{-5}	0.8713	0.023 (0.014)	0.0005	0.2384	0.019 (90.012)	0.0010	0.3511
Other CDI, no. cases/100 OBD	0.015 (0.012)	0.0001	0.7454	0.018 (0.014)	0.0007	0.0597	0.021 (0.008)	–0.0001	0.8753
HA-MRSA, no. cases/100 OBD	0.076 (0.035)	–0.0030	**0.0038**	0.076 (0.028)	0.0012	0.1588	0.051 (0.019)	–0.0004	0.8007
Other MRSA, no. cases/100 OBD	0.47 (0.077)	0.0047	0.0514	0.46 (0.082)	0.0008	0.7355	0.48 (0.08)	–0.0099	0.1444
New ESBL–producing organisms, no. cases/100 OBD	0.026 (0.014)	9.68×10^{-5}	0.8373	0.049 (0.029)	0.0006	0.5071	0.083 (0.021)	–0.00208	0.2441
Chlorhexidine skin wash, L/100 OBD	1.29 (0.261)	–0.0069	0.4109	1.15 (0.117)	–0.0056	0.1038	1.16 (0.125)	–0.0034	0.7626
Alcohol-based hand rub, L/100 OBD	1.33 (0.337)	–0.0020	0.8574	1.31 (0.409)	0.0375	**0.0006**	1.28 (0.22)	0.0012	0.5344
Piperacillin/tazobactam use, DDDs/100 OBD	12.72 (1.559)	0.1119	**0.0184**	10.14 (3.485)	0.1211	0.2472	14.26 (1.12)	0.1471	0.1185
Macrolide use, DDDs/100 OBD	17.39 (4.797)	–0.0613	0.6942	12.96 (9.388)	–0.0126	0.9649	18.49 (3.21)	–0.1699	0.5521
Co-amoxiclav use, DDDs/100 OBD	20.76 (3.506)	0.3603	**0.0002**	18.49 (8.579)	0.1212	0.6422	21.65 (2.48)	0.2996	0.1564
Fluoroquinolone use, DDDs/100 OBD	2.71 (0.726)	0.0033	0.8883	10.39 (5.86)	–0.0534	0.7647	6.32 (1.64)	0.1486	0.3001
β-lactamase–sensitive penicillins_J01CE, DDDs/100 OBD	2.62 (0.55)	–0.002451	0.8912	2.2 (1.22)	–0.117074	**0.0003**	0.96 (0.32)	–0.029760	0.2914
β-lactamase–resistant penicillins_J01CF, DDDs/100 OBD	8.26 (1.66)	–0.066818	0.2073	11.52 (2.18)	0.017952	0.7865	7.22 (1.79)	–0.272214	0.0647
Penicillins with extended-spectrum_J01CA, DDDs/100 OBD	34.75 (6.49)	0.470198	**0.0172**	53.86 (8.79)	0.223252	0.4011	38.75(3.98)	–0.535384	0.1106
Monobactams, DDDs/100 OBD	1.36 (0.44)	0.023281	0.0957	6.48 (2.40)	–0.055678	0.4431	1.52 (1.55)	–0.238161	**0.0049**
Carbapenems, DDDs/100 OBD	4.00 (0.89)	–0.001640	0.9550	3.12 (1.02)	–0.112413	**<0.0001**	2.16 (0.48)	–0.024279	0.5687
1st-generation cephalosporins_J01DB, DDDs/100 OBD	0.43 (0.21)	0.021690	**0.0002**	0.84 (0.28)	–0.007065	0.3972	0.58 (0.10)	0.002397	0.7928
2nd-generation cephalosporins_J01DC, DDDs/100 OBD	0.05 (0.06)	0.000988	0.6156	0.37 (0.22)	0.011200	0.0831	0.27 (0.06)	0.000150	0.9790
3rd-generation cephalosporins_J01DD, DDDs/100 OBD	0.39 (0.18)	0.004219	0.4572	0.57 (0.36)	0.020622	**0.0474**	0.62 (0.39)	0.029161	0.4023
Tetracyclines_J01A, DDDs/100 OBD	11.6 (2.63)	0.129842	0.1187	21.76 (7.86)	0.124861	0.6014	11.79 (1.96)	0.006404	0.9711
Glycopeptides, DDDs/100 OBD	8.57 (1.61)	0.001779	0.9730	12.8 (3.23)	–0.018187	0.8534	8.85 (0.99)	0.003556	0.9682
Total antibiotic use, DDD/100 OBD	146.2 (13.26)	0.780613	0.0591	192.00 (16.96)	0.114352	0.8249	148.14 (8.77)	–0.345771	0.6594
Causeway Hospital									
HA-CDI, no. cases/100 OBD	0.030 (0.031)	–0.0009	0.3858	0.017 (0.014)	$–5.14 \times 10^{-4}$	0.2101	NR	NR	NR
Other CDI, no. cases/100 OBD	0.012 (0.012)	–0.0005	0.2350	0.016 (0.019)	9.65×10^{-5}	0.8705	NR	NR	NR
HA-MRSA, no. cases/100 OBD	0.076 (0.031)	–0.0008	0.4534	0.074 (0.035)	–0.0013	0.2150	NR	NR	NR
Other MRSA, no. cases/100 OBD	0.513 (0.119)	–0.0068	0.0654	0.522 (0.102)	0.0059	**0.0460**	NR	NR	NR
MRSA, no. screens/100 OBD	2.20 (0.16)	–0.0008	0.8703	2.56 (0.24)	0.0111	0.1231	NR	NR	NR
New ESBL-producing organisms, no. cases/100 OBD	0.036 (0.033)	0.0004	0.6741	0.038 (0.030)	0.0015	0.0793	NR	NR	NR
Chlorhexidine skin wash, L/100 OBD	1.95 (0.33)	–0.0249	**0.0360**	1.85 (0.22)	9.78×10^{-4}	0.8827	NR	NR	NR
Alcohol-based hand rub, L/100 OBD	NR	NR	NR*	1.10 (0.50)	–0.0056	0.7131	NR	NR	NR
Piperacillin/tazobactam use, DDDs/100 OBD	13.07 (1.37)	0.0370	0.4030	12.998 (2.068)	0.086	0.1630	NR	NR	NR
Macrolide use, DDDs/100 OBD	17.94 (4.32)	–0.1713	0.2154	17.389 (3.941)	0.0756	0.5272	NR	NR	NR
Co-amoxiclav use, DDDs/100 OBD	19.33 (5.08)	0.3848	**0.0122**	23.551 (3.129)	0.0056	0.9532	NR	NR	NR
Fluoroquinolone use, DDDs/100 OBD	3.89 (1.76)	–0.0629	0.2644	5.868 (2.371)	0.1916	**0.0035**	NR	NR	NR
β-lactamase–sensitive penicillins_J01CE, DDDs/100 OBD	1.92 (0.93)	–0.002451	0.8912	1.92 (1.56)	–0.107652	**0.0158**	NR	NR	NR
β-lactamase–resistant penicillins_J01CF, DDDs/100 OBD	6.82 (3.39)	–0.002451	0.8912	7.97 (1.98)	0.068039	0.2517	NR	NR	NR

Variable	Pre-intervention, 2011 Nov–2013 Sep			Intervention, 2013 Oct–2015 Sep			Postintervention, 2015 Oct–2016 Sep		
	Average use (SD)	Trend coefficient	p value	Average use (SD)	Trend	p value	Average incidence/use (SD)	Trend coefficient	p value
Penicillins with extended-spectrum_J01CA, DDDs/100 OBD	30.57 (5.71)	–0.113964	0.7151	35.71 (8.42)	0.708057	**0.0022**	NR	NR	NR
Monobactams, DDDs/100 OBD	0.91 (0.49)	0.028603	0.2951	1.86 (0.99)	0.033870	0.2530	NR	NR	NR
Carbapenems, DDDs/100 OBD	3.9 (1.39)	0.000882	0.9911	3.62 (1.44)	–0.106339	**0.0091**	NR	NR	NR
1st-generation cephalosporins_J01DB, DDDs/100 OBD	0.42 (0.32)	0.011632	0.5211	0.52 (0.24)	–0.014970	**0.0282**	NR	NR	NR
2nd-generation cephalosporins_J01DC, DDDs/100 OBD	0.22 (0.22)	–0.020279	0.0979	0.30 (0.17)	0.007696	0.1205	NR	NR	NR
3rd-generation cephalosporins_J01DD, DDDs/100 OBD	0.55 (0.31)	0.003441	0.8461	0.65 (0.46)	0.008809	0.5270	NR	NR	NR
Tetracyclines_J01A, DDDs/100 OBD	9.46 (2.5)	0.163824	0.2396	12.56 (4.7)	0.359609	**0.0063**	NR	NR	NR
Glycopeptides, DDDs/100 OBD	7.68 (2.23)	0.021618	0.8651	7.98 (1.84)	0.135931	**0.0089**	NR	NR	NR
Total antibiotic use, DDD/100 OBD	135.62 (19.24)	0.381676	0.7279	152.49 (19.45)	1.527800	**0.0048**	NR	NR	NR

*CDI, *Clostridiodes difficile* infection; DDDs, defined daily doses; HA, healthcare-associated; MRSA, methicillin-resistant *Staphylococcus aureus*; NR, data not recorded; OBD, occupied bed days. Bold type indicates statistical significance.

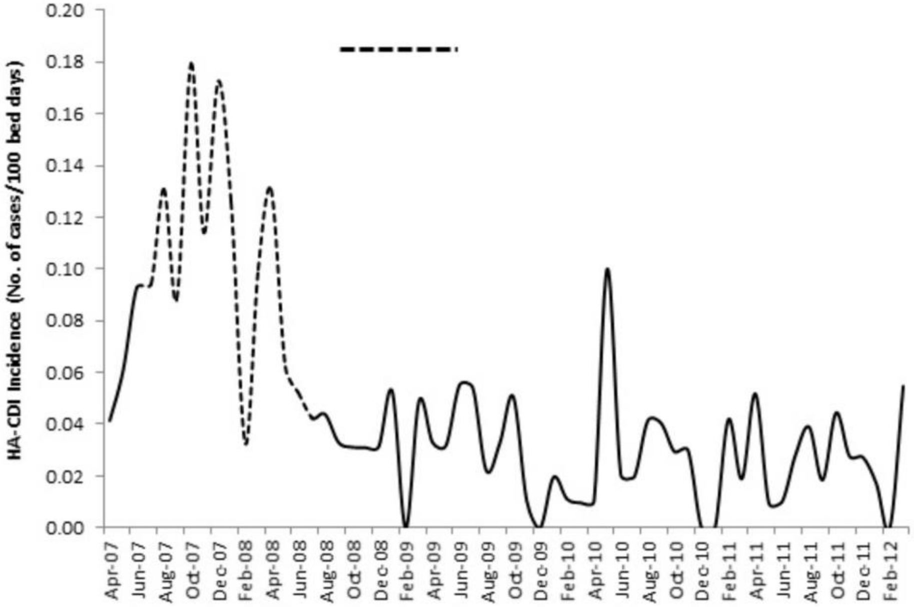

Appendix Figure 1. Incidence of healthcare-associated *Clostridiodes difficile* infection in Antrim Area Hospital, Northern Ireland, UK, April 2007–March 2012.

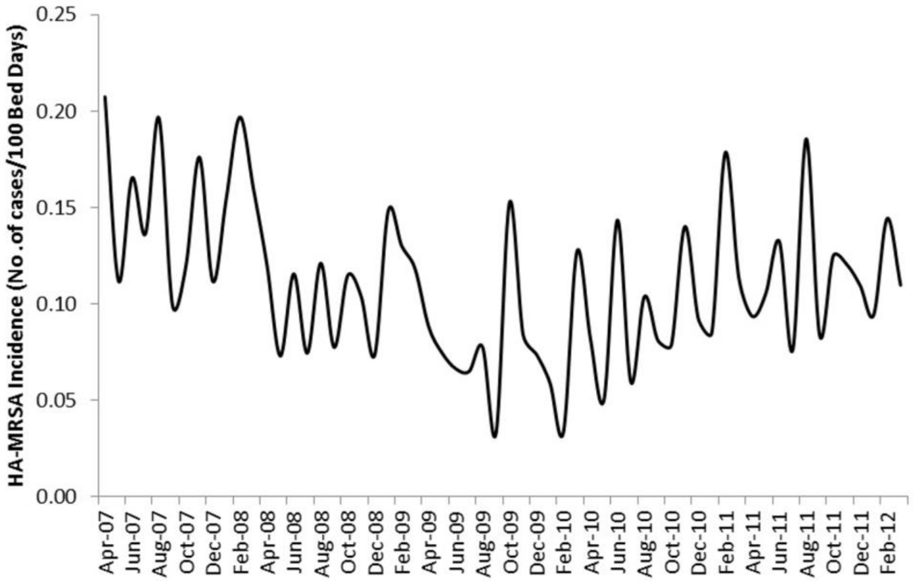

Appendix Figure 2. Total incidence of healthcare-associated methicillin-resistant *Staphylococcus aureus* in Antrim Area Hospital, Northern Ireland, UK, April 2007–March 2012.

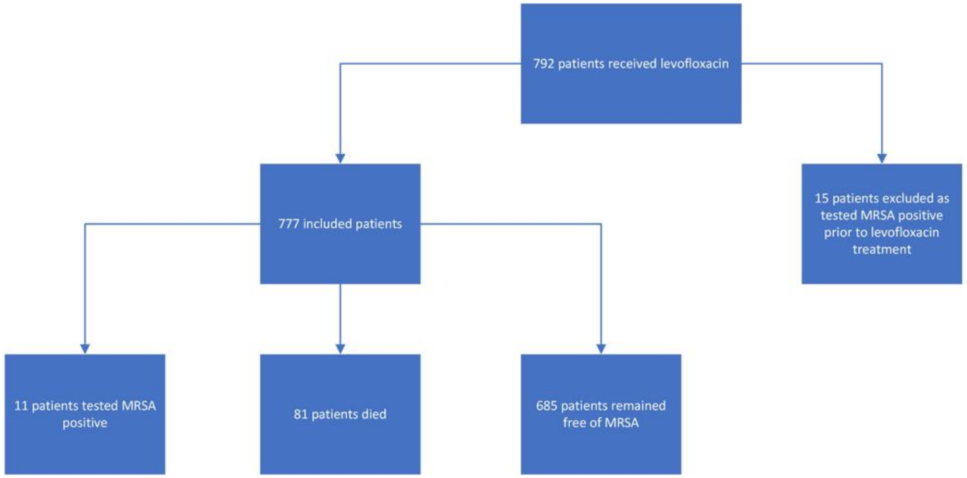

Appendix Figure 3. Methicillin-resistant *Staphylococcus aureus* acquisition in patients who received levofloxacin.

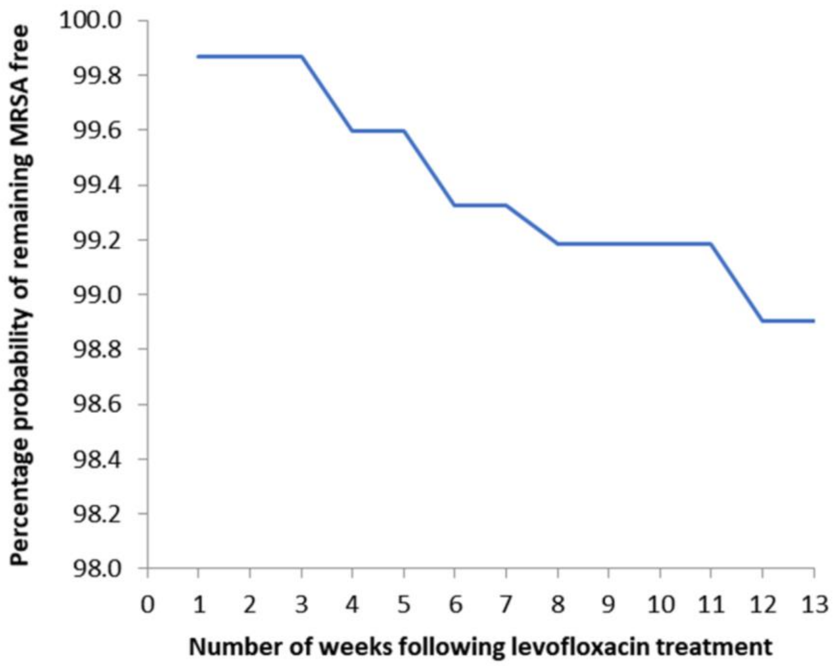

Appendix Figure 4. Percentage probability of remaining free of methicillin-resistant *Staphylococcus aureus* 12 weeks after levofloxacin treatment.

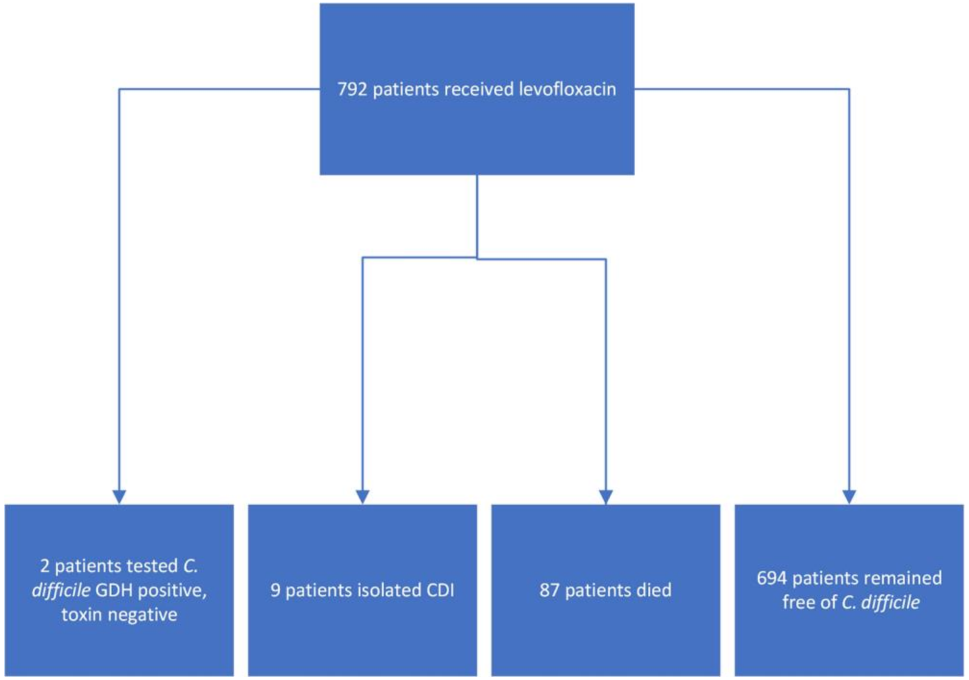

Appendix Figure 5. *Clostridioides difficile* infection acquisition in patients who received levofloxacin.

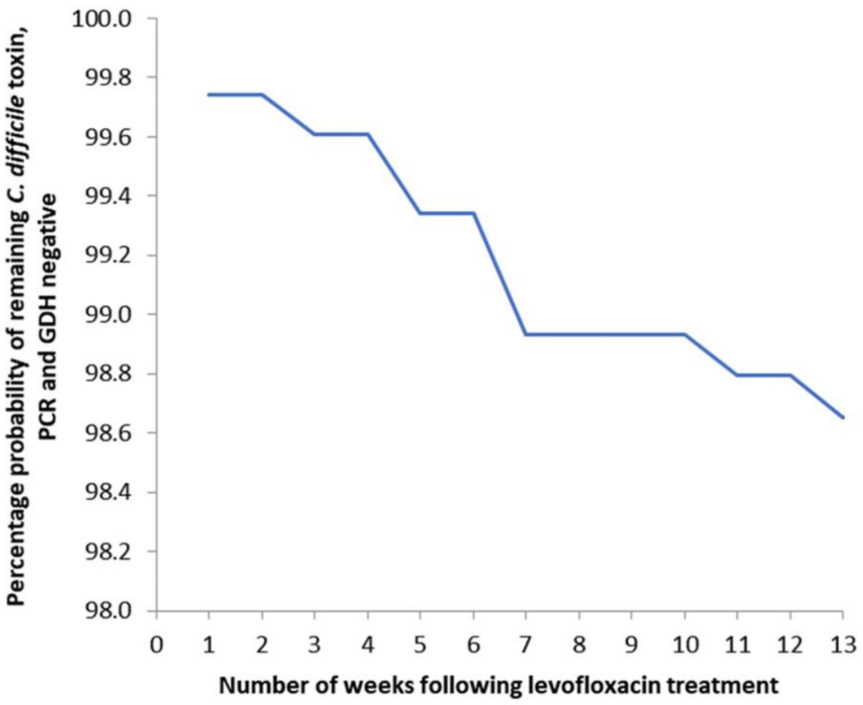

Appendix Figure 6. Percentage probability of remaining free of colonization or infection with *Clostridioides difficile* after levofloxacin treatment.
